# Economic evaluation of a reduced dosage of ready‐to‐use therapeutic foods to treat uncomplicated severe acute malnourished children aged 6–59 months in Burkina Faso

**DOI:** 10.1111/mcn.13118

**Published:** 2021-02-23

**Authors:** Dieynaba S. N'Diaye, Bibata Wassonguema, Victor Nikièma, Suvi T. Kangas, Cécile Salpéteur

**Affiliations:** ^1^ Research unit, Expertise & Advocacy Department Action Contre la Faim Paris France; ^2^ Action Contre la Faim Ouagadougou Burkina Faso; ^3^ Department of Nutrition, Exercise and Sports University of Copenhagen Copenhagen Denmark

**Keywords:** children, economic evaluation, outpatient care, ready‐to‐use therapeutic foods, severe acute malnutrition

## Abstract

Ready‐to‐use therapeutic foods (RUTF) used to treat children with severe acute malnutrition (SAM) are costly, and the prescribed dosage has not been optimized. The MANGO trial, implemented by Action Contre la Faim in Burkina Faso, proved the non‐inferiority of a reduced RUTF dosage in community‐based treatment of uncomplicated SAM. We performed a cost‐minimization analysis to assess the economic impact of transitioning from the standard to the reduced RUTF dose. We used a decision‐analytic model to simulate a cohort of 399 children/arm, aged 6–59 months and receiving SAM treatment. We adopted a societal perspective: direct medical costs (drugs, materials and staff time), non‐medical costs (caregiver expenses) and indirect costs (productivity loss) in 2017 international US dollar were included. Data were collected through interviews with 35 caregivers and 20 informants selected through deliberate sampling and the review trial financial documents. The overall treatment cost for 399 children/arm was $36,550 with the standard and $30,411 with the reduced dose, leading to $6,140 (16.8%) in cost savings ($15.43 saved/child treated). The cost/consultation was $11.6 and $9.6 in the standard and reduced arms, respectively, with RUTF accounting for 56.2% and 47.0% of the total. The savings/child treated was $11.4 in a scenario simulating the Burkinabè routine SAM treatment outside clinical trial settings. The reduced RUTF dose tested in the MANGO trial resulted in significant cost savings for SAM treatment. These results are useful for decision makers to estimate potential economic gains from an optimized SAM treatment protocol in Burkina Faso and similar contexts.

**Key messages**

- Ready‐to‐use therapeutic foods (RUTF) used to treat children with severe acute malnutrition (SAM) in community‐based setting are costly, and the prescribed dosage has not been optimized.
- The MANGO trial implemented in Burkina Faso demonstrated the non‐inferiority of a reduced RUTF dosage in community‐based treatment of uncomplicated SAM compared with the standard dosage.
- We conducted a cost‐minimization analysis alongside this trial, which has shown that reducing the dosage leads to 16.8% or $15.43 of cost savings per child treated.
- These results are useful for decision makers to estimate potential economic gains from an optimized SAM treatment protocol in Burkina Faso and similar contexts.

## 1 INTRODUCTION

Severe acute malnutrition (SAM) affects an estimated 14.3 million children under 5 years of age worldwide (UNICEF/WHO/The World Bank, 2020). In order to reach and treat as many acutely malnourished children as possible, the World Health Organization (WHO), the World Food Programme and UNICEF recommend a community‐based approach. It consists of treating SAM without medical complications at home with the help of ready‐to‐use therapeutic foods (RUTF): energy dense, micronutrient fortified pastes [World Health Organization; World Food Programme; United Nations System Standing Committee on Nutrition; UNICEF (Genève: Organisation mondiale de la santé), 2007, consulted on 29 April 2020, on https://www.who.int/nutrition/topics/statement_commbased_malnutrition/en/]. This treatment approach leads to higher coverage rates but remains resource intensive partly due to RUTF that has been previously estimated to represent between 24% to 44% of the total treatment costs (Bachmann, 2009; Isanaka et al., 2017; Puett et al., 2013; Wilford, Golden, & Walker, 2012).

A third of the RUTF world production is produced by Nutriset (Malaunay, France) with local productions in several African countries, which sometimes struggle to attain sufficient quality standards (Segrè, Liu, & Komrska, 2017). Therefore, the RUTF is usually imported, cleared through customs and distributed by UNICEF in conjunction with the local ministries of health and the health districts. This leads to high costs and multiplicity of actors that can create supply chain disruption threatening the treatment sustainability for countries with weak health systems and scarce resources. It reinforces external dependence of low‐ and middle‐income countries on donors to purchase the nutritional products (United Nations Standing Committee on Nutrition, 2011).

Furthermore, there is a lack of evidence regarding the RUTF optimal dosage during SAM treatment. A study conducted in Myanmar in 2015 showed a high recovery rate in children treated with a reduced dosage of RUTF following a stock‐out (James et al., 2015). The MANGO trial investigated the non‐inferiority of a reduced dosage of RUTF after the third week of treatment on the recovery of 801 children with SAM aged 6–59 months without medical complications. This randomized trial provided a rigorous assessment of the clinical impact of a reduced dose and presents an opportunity to assess its economic consequences as well.

To our knowledge, no economic evaluation of a reduced dosage of the RUTF within the SAM outpatient treatment protocol has been undertaken. Hypothesizing that a reduced dose would result in considerable savings that will allow countries to better support the economic burden of SAM treatment, we aimed to perform an economic evaluation alongside the MANGO clinical trial. We seek to quantify the economic impact of the transition from the standard RUTF dose to the new reduced dose tested in the trial.

## 2 METHODS

### 2.1 Data and sources

#### 2.1.1 Settings and population

The MANGO trial was conducted between October 2016 and July 2018 in the health district of Fada N'Gourma in east of Burkina Faso where the non‐governmental organization (NGO) Action Contre la Faim (ACF) intervenes through its nutrition programme. The district has 48 health centres, 10 of them were chosen to participate in the study based on the number of SAM children registered per month (minimum of seven new SAM cases per month), their accessibility and a suitable schedule to couple study visit days with routine growth monitoring days. The children included in the study are those aged 6 to 59 months who in accordance to the national protocol for SAM management in Burkina Faso had a weight‐for‐height *z* score <−3 and/or a mid‐upper arm circumference (MUAC) < 115 mm, no oedema, who passed an appetite test and presented no medical complications. Other exclusion criteria included children having received SAM treatment in the last 6 months and caregiver planning to travel or unable to comply with the weekly check‐up schedule, peanut or milk allergy or disability affecting food intake (Kangas et al., 2019).

#### 2.1.2 MANGO protocol

The children included in the MANGO trial were treated with strict compliance to the national protocol for SAM management in Burkina Faso (Burkina Faso Ministry of Health, 2014). After admission, children were received for weekly follow‐up consultations until discharge. Children could be discharged from the study as recovered (reaching recovery anthropometric criteria), referred (presenting medical complications, having more than 5% weight loss within 3 weeks and having no more than 100‐g weight gain over 4 weeks), defaulted (missing three consecutive visits but confirmed alive or transferred to health centres not involved in the study), non‐responder (still not attaining recovery criteria by 16 weeks of treatment) or died. Each consultation consisted of the study staff taking the child's anthropometric measurements and a nurse performing a clinical examination to identify co‐morbidities and potential medical complications and finally, if the child passed the appetite test, prescribing RUTF according to the weight, treatment week and trial arm of the child. The caregivers were also provided sensitization messages on child feeding and the use of the RUTF. As part of SAM treatment, children received systematic medical treatment consisting of the administration of an antibiotic (amoxicillin) on the day of admission, anthelmintic (albendazole) at the second visit and antimalarial drugs (arthemeter + lumefantrine) at any visit if the malaria rapid diagnostic test was positive. During the clinical examination, any diagnosed illnesses like malaria, diarrhoea and acute respiratory infections were also treated. Children who presented complications (hypoglycaemia, acute dehydration, septic shock, gastric dilation, heart failure, hypothermia, severe anaemia, fever and corneal ulceration) during a visit were referred for inpatient care and did not return in the trial. As per national protocol, children who missed a programmed visit were contacted by telephone or actively searched in the community by the community health workers to encourage them to continue the weekly follow‐up visits.

#### 2.1.3 Perspective

As this study aims to assess the economic impact of a change of protocol for Burkinabe health decision makers, a societal perspective was preferred. It includes the costs borne by ACF and its partners, the health system and communities (Russell, Fryback, & Sonnenberg, 1999; Weinstein, Siegel, Gold, Kamlet, & Russell, 1996). The portion of global costs borne only by institutions (excluding community costs) was also assessed.

#### 2.1.4 Strategies compared

A total of 402 children with SAM were treated with standard RUTF dose (control arm) and 399 children with reduced RUTF dose (intervention arm). In order to be able to make a comparison based on two arms with identical number of participants, we randomly removed three children from the arm that received the standard dose. We therefore performed the economic evaluation with 399 children per arm.

Socio‐economic characteristics of children recruited in the two arms were not different (Kangas et al., 2019). All children were treated in accordance with the national community‐based management of acute malnutrition (CMAM) protocol (Ministère de la santé BF, 2014). However, in the intervention group, children received a reduced dose of RUTF from the third week of treatment onwards, prescribed according to the weight of the child (see Table 1).

**TABLE 1:** RUTF dosage in the MANGO trial

	Standard RUTF dose	Reduced RUTF dose		Percent of reduction between the standard and reduced dose
	Admission to discharge	Weeks 1–2	Week 3 to discharge	From Weeks 1–2 to Week 3
Weight (kg)	Sachets/week			
3.0–3.4	8	8	7	13
3.5–4.9	10	10	7	30
5.0–6.9	15	15	7	53
7.0–9.9	20	20	14	30
10.0–14.9	30	30	14	53

Abbreviation: RUTF, ready‐to‐use therapeutic food.

The RUTF used during the trial came from three different sources and had different supply methods and pipeline. Fifty percent of the RUTF came from the health district stock, which was purchased by UNICEF. The remaining RUTF came from the MANGO buffer stock directly purchased by the trial investigators to prevent stock‐outs during the study. This buffer was composed of RUTF from Nutriset (60%) France, air cargoed to Burkina, and RUTF from InnoFaso (40%), a local RUTF manufacturer in Burkina Faso.

#### 2.1.5 Time horizon

The time horizon of the evaluation corresponds to the implementation period of the MANGO trial, that is, 2 years and 2 months between October 2016 and December 2018.

#### 2.1.6 Mango efficacy results and corresponding type of economic analysis

The MANGO efficacy results confirmed the non‐inferiority of the reduced dose in both intention to treat (inferiority rejected, *p* = 0.013) and per protocol (inferiority rejected, *p* = 0.019) for the main outcome, the weight gain velocity. The mean weight gain velocity from admission to discharge was 3.4 g/kg/day with no difference between the reduced and the standard arms [Δ 0.0 g/kg/day; 95% confidence interval (CI) −0.4 to 0.4; *p* = 0.92]. The recovery rate was similar in both arms (52.7% and 55.4%; *p* = 0.45), and there were no significant difference on the length of stay (median = 56 days). The percent of referrals, defaulters, non‐responses and relapses were not statistically different between the reduced and the standard arms (Kangas et al., 2019). As recommended for economic evaluations alongside equivalence trials, we conducted a cost‐minimization analysis (Briggs & O'Brien, 2001).

#### 2.1.7 Outcomes

Only cost outcomes were considered. We included the direct medical costs, direct non‐medical costs and indirect costs. The direct medical costs include costs related to medical staff time, material, consumables, drugs and RUTF (related purchase, international shipping, national transport and management costs) used from treatment admission until discharge. The RUTF management costs include cost related to storage, guarding and daily management (temperature monitoring, expiration dates checking process, exchanges with the districts, weekly briefings with ACF pharmacist and the district nurse). The direct non‐medical costs are related to transport and other out‐of‐pocket expenses supported by the beneficiaries while seeking SAM treatment. Indirect costs are defined as costs related to temporary absence from work due to illness, reduced working capacity due to illness and disability or lost productivity due to early death (Kirch, 2008). Further details on each cost component are available in the technical appendix. The evaluation criterion was the difference between the costs involved in each arm.

#### 2.1.8 Data collection

Data used in this study were collected through two data field collections performed in 2017, from 17 to 27 October and again from 26 November to 8 December in both Ouagadougou and the Fada N'Gourma health district. A mapping of activities and resources used in the MANGO project was established based on in‐depth interviews and observation during the enrolment phase of the trial. Relevant data to estimate costs were then collected via semi‐structured interviews and focus group discussions with key informants and beneficiaries. In total, 35 caregivers of children enrolled and more than 20 other key informants were selected through deliberate sampling. Members of the MANGO team, key staff members of ACF offices in both Fada N'Gourma and Ouagadougou and relevant stakeholders of the Burkinabé health system (community health workers and hospital and health centre staff) were administrated in‐person questionnaire on the allocation of time and resources used as well potential relevant data sources. Seventeen face‐to‐face semi‐structured interviews were conducted as well as three focus group discussions and structured observations in 2017 in 8 out of 10 health centres participating in the trial. Responses were triangulated with different interviewees to minimize bias. Further information on data collection can be found in the appendix.

#### 2.1.9 Cost estimation and analysis

The costs for each step in the trial were proportionally allocated based on actual usage during the implementation. When possible, an ingredients‐based approach was used to estimate unit costs. Following the guidelines, all resources that may substantially influence overall costs were collected and all big item costs that are differential between the two arms were assessed (Ramsey et al., 2015).

This study does not include the costs that had no impact on the intervention itself such as research costs (team supervisors, research manager and principal investigator salaries, research trainings and research material). We only considered prospective costs incurred as part of this study and focused on costs involved in SAM treatment (from the admission to discharge and cost to track absent or defaulting children by community health workers). Thus, all the costs related to routine practice but not associated with outpatient treatment (screening in the community, referral to inpatient or other Outpatient Therapeutic Program (OTPs) and SAM treatment training) were excluded. Costs related to post‐discharge consultations and inpatient care after referrals were also excluded. All infrastructure costs (buildings, electricity, running water, etc.) were considered irrelevant to the research question and were not collected.

Costs for human resources involved in each step of the trial were calculated based on the estimated time required to perform their activities, using time estimation questionnaires, and salary value for their position.

Material costs used in the follow‐up consultations included those associated to the use of the thermometers, stethoscope, height boards, weight scales and transportation boxes and were annuitized over their expected lifetime to calculate their depreciation value for their use during the project (Drummond, Sculpher, Claxton, Stoddart, & Torrance, 2015; Johns, Baltussen, & Hutubessy, 2003). The value of all the consumables (used routinely during treatment consultations) was related to the total number of consultations in order to have the equivalent for a single consultation. Trial consumables included, but were not limited to, hand disinfectant and sanitizer, disposable gloves, cotton rolls, disinfectant for height boards, MUAC tapes, office supplies and telephonic credit cards reserved for the missed weekly visits management. The costs of the consumables related to research activities were excluded.

The costs borne by the communities included the beneficiaries' out‐of‐pocket expenses and the costs related to the support of the community health workers. The out‐of‐pocket expenses included travel and food costs borne the day of the consultation by each beneficiary and were assessed via face‐to‐face caregiver interviews. Caregiver's loss of productivity (or opportunity cost) was calculated as the potential income lost due to the follow‐up consultation, as if they had otherwise performed an activity with an economic value (Drummond et al., 2015; Puett et al., 2013). Caregiver characteristics collected in the baseline questionnaires of the trial showed that 95% reported ‘housewife’ as their status, and therefore, housework was considered as their main occupation. Opportunity cost for the household was based on the average wage the household would have paid for domestic help using Burkina Faso mandatory minimum wage for such occupation [Commission Mixte Paritaire de Négociations Salariales du Secteur Privé (CMPNSSP), 2012]. While being aware of the fact that households would not be able to hire domestic help, we aimed to estimate the market value of the domestic work that could not be performed due to the MANGO consultation. For the community health workers, the economic value of the activities as part of the trial, which consisted in searching for children who missed their weekly visits, was estimated using the same method as for the beneficiaries.

#### 2.1.10 Model overview

**FIGURE 1:**
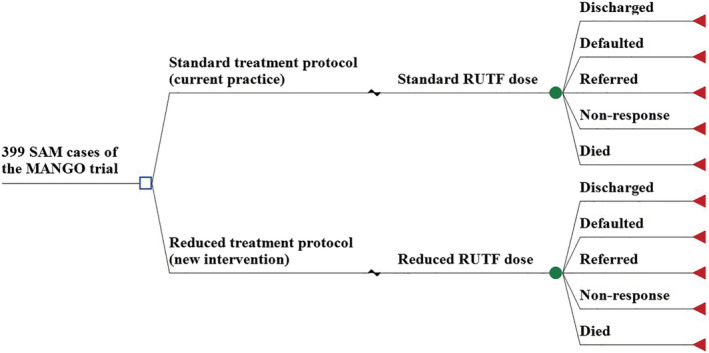
Representation of the decision tree model. RUTF, ready‐to‐use therapeutic food; SAM, severe acute malnutrition. A defaulter was defined as child that missed three consecutive visits, but who was confirmed to be alive. Referrals included children referred to inpatient care as a result of medical complications. Non‐responder applied to children still not attaining recovery criteria by 16 weeks of treatment (Kangas et al., 2019)

The unit costs estimated as described above were then used as parameters in a decision tree model simulating the trajectory of all SAM children included in the MANGO trial. The model followed each child in the cohort from their enrolment until discharge comparing both groups (see Figure 1). Each event modelled is associated with its cost in both treatment strategies in the Burkina Faso context. Total costs of the compared interventions were calculated based on the sum of each unit cost calculated for the nutrition follow‐up consultation (weekly visits), adjusted to reflect the proportion of individuals who actually experienced a follow‐up visit in each arm of the MANGO trial.

The cost data were cleaned and analysed with Microsoft Excel 2016 and Stata version 15 software, and the decision analysis model was developed with the TreeAge software (TreeAge Pro 2017, Health Care Edition, Williamstown, MA). Costs were reported as per consultation. Total costs of each arm were presented overall, by type of cost (direct medical, non‐medical and indirect costs) and over the major or relevant expense items (RUTF, drugs, materials, consumables, human resources and communities).

As recommended, no discounting nor inflation adjustments were applied as most of the costs incurred and were measured in the same 2017 year (Turner, Lauer, Tran, Teerawattananon, & Jit, 2019). The costs that were expressed in franc of the Financial Community of Africa (CFA) were initially converted into euro at the rate of 1 euro = 655.96 CFA before being converted into the 2017 international dollar using purchasing power parities.

### 2.2 Sensitivity analysis

#### 2.2.1 Univariate sensitivity analysis

We performed a univariate sensitivity analysis by varying the cost parameters individually over a range of plausible values to identify major cost drivers. The minimum and the maximum values were estimated based on data collected, assumptions and expert opinions. The impact of considering an identical value between the trial arms was also explored. Details on these values and the different assumptions made can be found in the technical appendix.

#### 2.2.2 Multivariate sensitivity analysis

We conducted multivariate sensitivity analysis whereby various parameters of the model were varied simultaneously to assess the joint uncertainty of all the model parameters over different scenarios.

First, a worst and best case scenario were explored to assess the minimum and the maximum saving incurred by the reduced dosage. They combined the estimated minimum and maximum values used in the univariate sensitivity analysis, respectively.

Second, we performed a ‘real‐life’ scenario reflecting the Burkinabé context. Indeed, during the MANGO trial, additional costs that would not occur in real‐life practice were generated mainly relating to human resource costs, drugs and consumables. Furthermore, a buffer stock of RUTF was put in place to compensate for possible RUTF shortages during the trial, and drugs and medical consumables were mainly purchased on the international market. Therefore, in this ‘real‐life’ scenario where the intervention is imagined to be delivered by routine staff via routine services, the salaries were indexed to local wages (rather than international NGO standards), consultations were made shorter, RUTF and consumables were assumed to be purchased locally and, consequently, no international transport costs were included. Regarding the materials used, local health workers estimated that equipment were used longer in real life (we assumed +25% on the normal time of use), so a smaller total depreciation charge was considered.

### 2.3 Ethical considerations

This study has been approved by the Burkina Faso Ethics Committee of Health Research. Participants enrolled in the economic evaluation were caregivers of children included in the MANGO trial, the research study teams from ACF in Burkina Faso and key individual informants from the health system. The informed consent for the MANGO study included an explanation on collection of economic information at the trial's inclusion. Another consent form specific for the economic evaluation was administrated to the 35 caregivers interviewed for further data collection. Informed consent to participate in the study was obtained from participants prior to any data collection. Permission to record interviews was collected verbally from the participants and the necessary steps were taken to ensure their confidentiality. No incentives were provided to study participants.

## 3 RESULTS

### 3.1 Base case results

The patient's socio‐demographic and clinical characteristics were previously reported in the trial efficacy paper (Kangas et al., 2019). Children in both arms were on average 13 months of age at admission. Only 14% and 15% of the beneficiaries were living in an urban area, 63% in the reduced arm and 61% reduced dosage arms lived more than 30‐min return trip to the health centre, respectively.

The children with SAM treated under the reduced dose protocol had more follow‐up consultation visits (3,174 consultations) compared with children treated under the standard dose protocol (3,163 consultations) even though statistical analysis showed that the length of stay was not significantly different between the two groups. The waiting time [3.5 h (2–5.7)] was longer than the consultation time itself [0.7 h (0.1–1.1)]. Logically, there was no difference in terms of waiting and consultation time between the two arms as the only difference between arms is the number of RUTF sachets given.

The main unit costs are presented in Table 2 and a full list can be seen in the appendix. The average cost per sachet prescribed during the trial was $0.33, the average cost of drugs delivered per consultation was $0.2 and human resource cost was $1.9 per treatment consultation. The average total costs per treatment consultation was $11.6 and $9.6 in the standard and reduced arms, respectively, with RUTF and related transport and storage costs accounting for 56% and 47% of the total consultation.

**TABLE 2:** Unit costs and quantities used as model parameters: Base case values, assumptions and ranges used in the sensitivity analyses

Parameters	Description	Base case value	Assumptions for min values	Assumptions for max values	Sources
Cohort size	a = Total number of children included in the trial and considered for the analysis	798	NA	NA	Trial database
Total number of treatment consultations	b = c + d	6,337	NA	NA	
Standard protocol	c = Total number of treatment consultations in the standard dosage arm	3,163	NA	NA	Trial database
Reduced protocol	d = Total number of treatment consultations in the reduced dosage arm	3,174	NA	NA	Trial database
Number of sachets prescribed during the trial	e = f + g		NA	NA	
Standard protocol	f = Number of RUTF sachets prescribed in the standard arm	61,580	NA	NA	Trial database
Reduced protocol	g = Number of RUTF sachets prescribedin the reduced dosage arm	42,839	NA	NA	Trial database
Cost of RUTF sachet	h = i + j = Average cost of the RUTF sachet taking into account the origin	$0.33 [0.25–0.49]	We assumed all the RUTF sachets were bought by the health system through its usual circuit (less expensive, purchased to Nutriset or InnoFaso through UNICEF)	We assumed all the RUTF sachet prescribed were directly bought to Nutriset (most expensive circuit)	Trial database and financial documents
Sachet	i = Amount paid to the producer	$0.28 [0.24–0.36]			
Management	j = Cost of transport from the capital to the implementation base and costs related to stock management	$0.06 [0.01–0.12]			
Cost of drugs^a^			We assumed that the drugs were purchased locally and took away transport and transit costs	We increased by 10% the value of the base case	Trial database for the quantities Financial documents for the costs
Standard protocol	Average cost of drugs prescribed during a treatment consultation in the standard arm	$0.22 [0.14–0.24]			
Reduced protocol	Average cost of drugs prescribed during a treatment consultation in the reduced arm	$0.24 [0.14–0.26]			
Material costs	Annuitized material costs over the project duration, equivalent for one consultation	$0.06 [0.05–0.08]	−25% was applied to the equipment useful time	+25% was applied to the equipment useful time	Interviews with local health workers and trial financial documents
Consumables costs Standard protocol Reduced protocol	Cost related to the consumables used for a single treatment consultation in the standard arm Cost related to the consumables used for a single treatment consultation in the standard arm	$0.92 [0.83–1.02] $0.93 [0.84–1.02]	−10% was applied on each consumable purchase cost	10% was applied on each consumable purchase cost	Trial financial documents
Human resource costs	Cost related to the time spent by health care workers to perform the treatment activities during a single consultation	$1.85 [0.3–3.5]	We used the time spent to perform the different treatment activities in the real life	We used the time estimated by the trail staff (these estimations were overvalued)	Interviews with the trial staff and use of the trial financial documents
Caregiver cost	K = L + M + P = Costs borne by the caregivers for a single consultation	$1.99 [1.18–2.93]			
Out‐of‐pocket expense (transport cost + food)	L = Expenses borne by caregivers for a consultation, mainly food purchase expenses	$0.68 [0.41–0.92]	We used the min of the range values of the confidence intervals of the mean calculated with the trial database	We used the max of the range values of the confidence intervals of the mean calculated with the trial database	Interviews with caregivers
Income loss	M = N × O = Income lost for seeking care	$1.31 [0.77–2.01]	NA	NA	
Time spent by the caregivers for a consultation (see technical appendix)	N = Return trip duration + time spent at the health care centre for a consultation	$5.04 [2.94–7.72]	We used the min values of the times communicated by the beneficiaries and the staff during the interviews.	We used the max values of the times communicated by the beneficiaries and the staff during the interviews.	Interviews with caregivers
Mandatory minimum monthly wage for domestic workers	O = Legal local minimum monthly wage for domestic workers	$45.35	NA	NA	Country legislation
Local hourly wage	O/174	$0.261	NA	NA	
Community health workers cost	Q = Average cost of the community health workers searching children who missed their consultations, equivalent for one consultation	$0.0027 [0.008–0.0046]	We used the min values of the times communicated by the community health workers during the interviews	We used the max values of the times communicated by the community health workers during the interviews	Interviews with community health workers and country legislation

Abbreviations: NA, not applicable; RUTF, ready‐to‐use therapeutic food.

^a^ Includes arthemether, amoxiciline and albendazole.

Table 3 presents the trial costs by main cost components. The overall treatment cost for the 399 children treated per arm was $36,550 in the standard dosage arm and $30,411 in the reduced dose arm, leading to a net cost savings of 16.8% over standard dosage treatment protocol. The cost per child treated was $91.6 and $76.2 in the standard and reduced dosage arms, respectively, leading to a cost saving of $15.4 per child treated in the MANGO trial. Cost reduction by child recovered could not be compared between the arms because the number of recovered children were not identical.

**TABLE 3:** Global treatment costs, base case analysis, expressed in 2017 dollar using purchasing power parity (1 euro = USD $0.80 PPP)

Cost components	Estimates over 25‐month period for 399 children per arm				
	Standard dose		Reduced dose		Savings/losses
**RUTF**	**20,558**	**(56.2%)**	**14,302**	**(47%)**	**−6,257**
**Supply**	**17,019**	**(46.6%)**	**11,840**	**(38.9%)**	**−5,180**
**Logistic and management**	**3,539**	**(9.7%)**	**2,462**	**(8.1%)**	**−1,077**
**Drugs**	**702**	**(1.9%)**	**746**	**(2.5%)**	**44**
**Consumables**	**2,923**	**(8%)**	**2,953**	**(9.7%)**	**30**
**Material**	**188**	**(0.5%)**	**189**	**(0.6%)**	**1**
**Human resources**	**5,864**	**(16%)**	**5,885**	**(19.4%)**	**20**
**Communities cost**	**6,315**	**(17.3%)**	**6,337**	**(20.8%)**	**22**
**Out‐of‐pocket expenses**	**2,151**	**(5.9%)**	**2,158**	**(7.1%)**	**7**
**Caregiver's opportunity cost**	**4,156**	**(11.4%)**	**4,170**	**(13.7%)**	**14**
**Community health workers' opportunity cost**	**9**	**(0.02%)**	**9**	**(0.03%)**	**0**
**Global costs of treatment consultations (institutional perspective)**	**30,235**		**24,074**		**−6,161**
**Cost per child treated (institutional perspective)**	**75.78**		**60.34**		**−15.68**
**Global costs of treatment consultations (societal perspective^#^)**	**36,550**	**(100%)**	**30,411**	**(100%)**	**−6,140**
**Cost per child treated (societal perspective^#^)**	**91.61**		**76.22**		**15.39**

*Note*. #The institutional perspective includes cost borne by Action Against Hunger and the health system. The societal perspective includes institutional perspective costs and community costs.

Abbreviation: RUTF, ready‐to‐use therapeutic food.

The principal source of savings induced by the reduced dosage was lower RUTF costs. These savings offset the marginal additional costs incurred for additional follow‐up consultations in the reduced arm.

The costs related to the human resources involved in providing care represent 16.0% and 19.4% of the global cost for the standard and the reduced dosage arms, respectively. The costs supported by the community were $2.0 per consultation and $15.9 for the average length of stay under treatment. In both arms, a third of community costs corresponded to out‐of‐pocket expenses from the families of children with SAM, whereas the rest corresponded to opportunity costs for both the caregivers and community health care workers. Community opportunity costs were equivalent to 11.4% and 13.7% of the overall treatment cost in the standard and the reduced dosage arms, respectively.

### 3.2 Sensitivity analysis results

#### 3.2.1 Univariate

**FIGURE 2:**
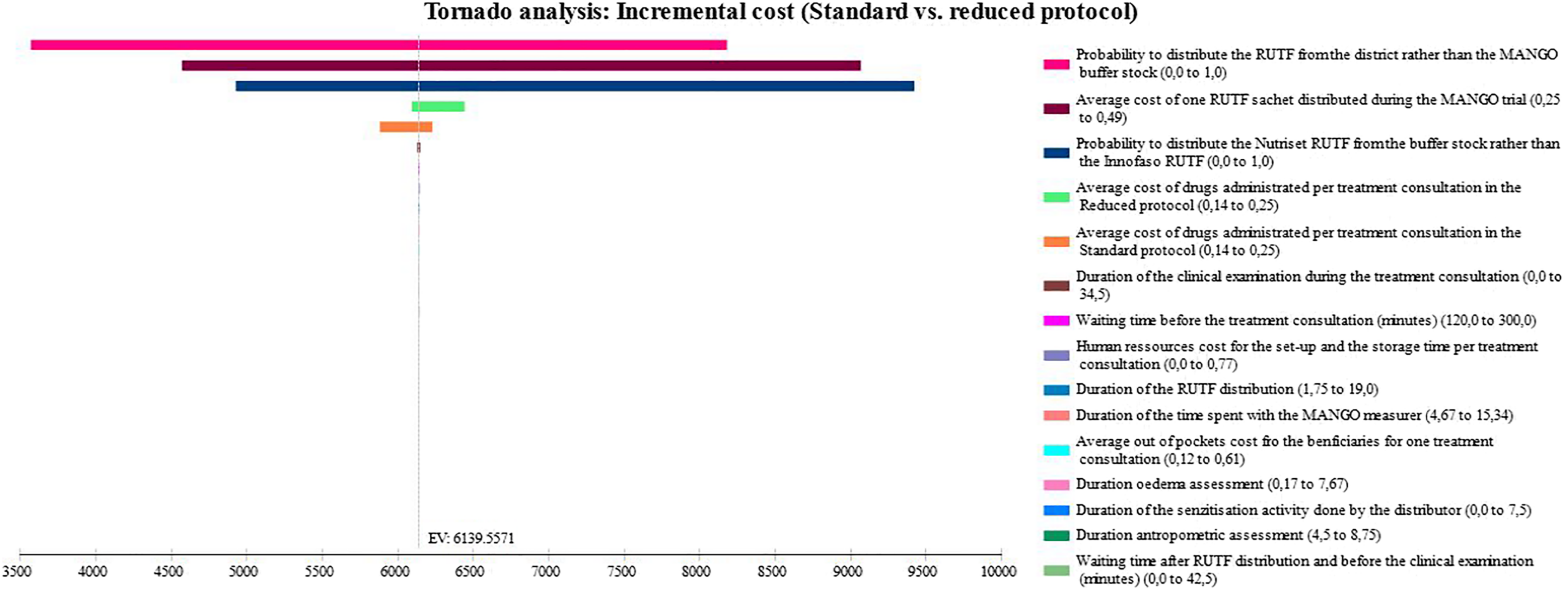
Univariate sensitivity analysis: Tornado diagram of global incremental cost between arms. The horizontal axis is our main outcome (the cost reduction between the compared protocols); along the vertical axis, parameters are arrayed, and horizontal bars represent the outcome range associated with each specified parameter's tested range in the sensitivity analysis. The outcome point estimate corresponding to base case values is indicated by the vertical line cutting through all horizontal bars. The two most influential variables on the difference between the two arms were the provenance of RUTF then followed by the average cost of drug administrated during a treatment consultation. Other variables had limited impact on incremental cost between the two dosages. RUTF: ready‐to‐use therapeutic food

A tornado analysis was performed to identify the key cost drivers. Results showed that main cost drivers were related to RUTF cost per dose and sourcing. Most influential variables on the cost per consultation were the same in both arms and included the provenance of RUTF prescribed, the cost of RUTF sachet, the duration of the clinical examination and beneficiaries' waiting time during the consultation. In both arms, community costs per consultation were mainly impacted by the waiting time before and during the consultation and beneficiaries' expenses (mainly food) during the treatment consultation. Global cost variations between the two protocols were mainly influenced by the provenance of RUTF prescribed, the cost per sachet and the amount of drug administrated in each arm (see Figure 2). When considering an identical number of consultations between the arms, transitioning towards the reduced dosage led to $6,192 in cost savings (vs. $6,140 in the base case). Further results can be found in the technical appendix.

#### 3.2.2 Multivariate

In the worst and best case scenarios, the global cost reduction between the two protocols ranged from 16.7% to 20.2% corresponding to savings per child treated between $11.4 and 22.5$. The ‘real‐life’ scenario showed a lower cost per consultation: $7.7 for the standard dose and $6.2 for the reduced one. Under the assumption of that scenario, global costs were $24,322 for the standard arm and $19,762 for the reduced one. It led to a 19% cost reduction corresponding to $11.4 per child treated of (vs. $15.4 in the base case). Further results of the sensitivity analysis can be found in the technical appendix.

## 4 DISCUSSION

Previous results of the MANGO trial showed that a reduced RUTF dose did not affect children's recovery from SAM, their length of stay, their vitamin A or iron status nor their body composition (Kangas et al., 2019; Kangas, Kaestel, et al., 2020; Kangas, Salpéteur, et al., 2020). Our study demonstrates that it also leads to significant cost savings. Reducing RUTF dosage from the third week onwards resulted in approximately $6,140 or 16.8% of cost savings for 399 cases with SAM included in each arm in the MANGO trial and $15.4 per child treated. To our knowledge, we are the first to perform an economic evaluation of a simplified SAM treatment only based on RUTF dose reduction; therefore, this estimation cannot be directly compared with those of other studies. Another trial, the ComPAS, tested a simplified SAM treatment protocol based on MUAC only as admission criteria, a reduced dosage of RUTF for different levels of severity and single product to treat both moderate and severe acute malnutrition in two countries in East Africa (Bailey et al., 2018). Cost reduction only related to dosage reduction cannot be pieced out of the trial results for comparison with our study. Authors found that their combined protocol costs $16 less per child treated (4% cost reduction) and US$123 less per child recovered compared with their standard protocol (using MUAC as admission criteria). However, the added costs of running a research trial could not be separated from treatment programme costs nor disaggregated by country, which hampers comparability.

We found that the cost per child treated was $91.61 and $76.22 in the standard and reduced protocols, respectively. Although hardly comparable because of different cohort characteristics, our results were in the same range as those reported in the literature for community‐based treatment of uncomplicated SAM in Africa. Indeed, when converted to 2017 dollars using purchasing power parity, the cost per child treated was $96 in the study by Isanaka et al. (2017) in Niger undertaken from a provider perspective that did not include household cost, $161 in Ethiopia that included institutional costs (Tekeste, Wondafrash, Azene, & Deribe, 2012) and $203 in Malawi from a health service perspective including hospitalization component of CMAM and capital cost (Wilford et al., 2012).

As in the other studies, the largest cost driver in the MANGO trial was the RUTF, but our results highlighted that the sourcing of the product was very influential. The product accounted for 56.2% and 47.0% of overall costs in the standard and reduced dosage arms, respectively. This proportion is higher than that reported by Isanaka et al. (2017) in Niger (44%). The RUTF cost estimations vary depending on whether or not storage and product management costs (that are rarely taken into account) are included. Its chosen mode of transportation affects its cost (airfreight being expensive compared with shipping) as well as whether international transportation is needed. Therefore, the RUTF share in the treatment costs depends largely on the RUTF supply chain in the country concerned. In the MANGO trial, only 17% of the prescribed RUTF was locally supplied, and it was 26% cheaper than the one supplied abroad. If all the RUTF used had been supplied through the Ministry of Health's usual supply circuit—which is the cheapest—the RUTF would have represented 48.6% of the cost per child treated with the standard protocol and lead to a cost reduction of 14% in the total consultation costs in that arm. In our study, the locally produced RUTF was cheaper than the one bought abroad and sent by air, but this may not be the case in other contexts. Indeed, producing RUTF locally can lead to very high production costs, especially when ingredients and manufacturing resources are rare and expensive. In low‐income countries, setting up a highly industrialized production system can be extremely costly. In addition, running costs of the production site (like electricity) can also lead to higher expenses that can then be reflected in the final product costs and make it more expensive than an imported product.

Our study further shed light on the importance of the costs borne by the community seeking treatment that are largely assumed to be free of charge in Burkina Faso. These costs for the household represented $2 per consultation so $16 for the trial's average length of treatment. As a comparator, WHO estimates a cost of (international dollar, 2014) $82 the total expenditure on health per capita per year in this country. Approximately 66% of these costs correspond to the value of time spent seeking care, a time that could be used for income‐generating activities for a population belonging to a low socio‐economic category. Innovative strategies that would minimize the time spent by the caregivers to seek care would lower this burden and might reduce default rates.

The high retention rate of the MANGO trial led to negligible costs for search of missing children by the community health workers. However, costs borne by those actors might be more significant in routine programme and should be further documented.

Concerning generalizability, the sensitivity analysis showed that the percent of cost reduction could range from 16.7% to 22.2% across the various scenarios explored. The one that was simulated in real life with routine practice in Burkina Faso showed that the global cost reduction between the two dosages would likely be around 19% or $11.4 saved per treated child. Overhead costs, not included in our analysis because embedded in a clinical trial, will vary greatly according to who implements the protocol with a reduced dose. They should be considered if one wished to perform a costing analysis of reduced dosage programme in a real‐life context. The costs reduction could also further vary in other contexts depending on the SAM population mean age, disease severity, average length of stay in treatment and other characteristics. Real‐life effectiveness of the reduced protocol still remains to be assessed as it usually differs from efficacy in clinical settings (Singal, Higgins, & Waljee, 2014).

Our study has several strengths. It was carried out alongside an individually randomized clinical trial that demonstrated good internal validity (Kangas et al., 2019; Kangas, Kaestel, et al., 2020; Kangas, Salpéteur, et al., 2020) and followed a strict compliance with the SAM management protocol in Burkina. Economic data including societal costs were collected during the enrolment phase, lowering the risk of memory bias in the participants interviewed. Thanks to having access of the trial data, we were able to perform an exhaustive and disaggregated costing of consumables and drugs used at each consultation, reflecting actual usage of inputs in each arm. Finally, a societal perspective including both direct and indirect costs allowed us to further approximate costs borne by the community during SAM treatment.

However, our study has several limitations. The opportunity cost of seeking care for the caregivers may have been overestimated. First, because no published data were available, the valuation of time spent to seek care was based on the hourly wage prevailing in Burkina Faso for domestic workers, which may be overestimated, as this legislation is not always followed. This is a strong limitation, but the durations estimated can be more accurately valued in the future provided that reliable data become available. However, inversely, we considered that the costs of transport from homes to health centres were negligible and were not included. Second, the time allocated to the various activities was collected on the basis of recall; therefore, response bias may exist. Third, the number of staff, their wages and their strict compliance to the protocol steps were not reflective of real‐life practice and the trial‐associated costs were likely greater than in real life. However, this impacted both arms similarly and therefore was nullified in the cost difference calculation; it did not affect the final percentage of cost reduction. Furthermore, the impact of these limitations was extensively explored in the sensitivity analysis and did not significantly change our main results.

The prevalence of SAM in Burkina Faso was 2.1% in 2017 (Performance Monitoring and Accountability 2020, 2017), with SAM treatment coverage estimated at 44% by UNICEF (2018). Treating those children with SAM in Burkina Faso with the reduced dose protocol could lead to a cost reduction of $357,216, which represents 0.05% of the country's total expenditure on health (WHO, 2014) and could be used to treat 7,212 more SAM cases. These estimates would need to be further assessed through a budget impact analysis, but they are likely to represent interesting savings without lowering the current effectiveness of the SAM treatment.

## 5 CONCLUSION

Our cost‐minimization analysis shows that the adoption of a reduced dosage of RUTF in the treatment of SAM might lead to substantial cost saving in the Burkina Faso context. The resources saved may be more efficiently used for malnutrition prevention and treatment activities.

## CONFLICTS OF INTEREST

Dieynaba S. N'Diaye, Bibata Wassonguema, Victor Nikièma, Suvi T. Kangas and Cécile Salpéteur declare that they have no conflict of interest.

## CONTRIBUTIONS

DSND and CS conceived the research project. DSND collected data in the field, performed the data analysis, interpreted the results, wrote the first draft or the manuscript, revised subsequent drafts and coordinated the contributors. BW participated in additional data collection, performed the data cleaning and analysis, contributed to interpretation of results and wrote the first draft. STK, VN and CS contributed to interpretation of results and revised subsequent drafts. CS acquired the funding for the study.

## Supporting information


**Table S1:** Data collection
Table S2: Material component costs (expressed in international $ 2017 using purchase power parity)
Table S3: Consumables component costs (expressed in international $ 2017 using purchase power parity)
Table S4: Human resources component costs (expressed in international $ 2017 using purchase power parity)s
Table S5: Daily management costs of the RUTF from the buffer stock (expressed in international $ 2017 using purchase power parity)
Table S6: Average cost of the RUTF sachet (expressed in international $2017 using purchase power parity)
Table S7: Drugs component costs (expressed in international $ 2017 using purchase power parity)
Table S8: Community costs (expressed in international $ 2017 using purchase power parity)
Table S9: Values and assumptions made for the sensitivity analysis
Table 10: Costs of the scenarios explored in the sensitivity analysis (expressed in international $ 2017 using purchase power parity)
Table 11: Global and incremental costs of the standard vs reduced dosage in the base case and the scenario explored in the sensitivity analysis (expressed in international $ 2017 using purchase power parity)
Table 12: Cost per children and number of children that could be treated with a budget of $100,000 under each scenario explored in the sensivity analysis in both arms of the MANGO trial (expressed in international $ 2017 using purchase power parity)
Figure S1: Cost components for a single treatment consultation in the MANGO trial per study arm
Figure S2: Tornado analysis on cost per consultation (Standard dosage arm)
Figure S3: Tornado analysis on cost per consultation (Reduced dosage arm)
Figure S4: Tornado analysis community costs in the standard dosage arm
Figure S5: Tornado analysis community costs in the reduced dosage arm
